# Clinical Application of Octacalcium Phosphate/Gelatin Composite in Spherical Periacetabular Osteotomy: Two Case Reports

**DOI:** 10.7759/cureus.99413

**Published:** 2025-12-16

**Authors:** Hideki Fukuchi, Hidetatsu Tanaka, Yu Mori, Osamu Suzuki, Toshimi Aizawa

**Affiliations:** 1 Department of Orthopedic Surgery, Tohoku University Graduate School of Medicine, Sendai, JPN; 2 Division of Craniofacial Function Engineering, Tohoku University Graduate School of Dentistry, Sendai, JPN

**Keywords:** artificial bone substitutes, developmental dysplasia of the hip (ddh), octacalcium phosphate, periacetabular osteotomy, spherical periacetabular osteotomy

## Abstract

Autologous bone grafts remain the gold standard for bone defect repair but are limited by donor-site morbidity. Octacalcium phosphate/gelatin composite (OCP/Gel) has shown strong osteoconductivity and osteoinductivity in animal studies, yet clinical reports are lacking. Two patients with developmental dysplasia of the hip underwent spherical periacetabular osteotomy (SPO) using β-tricalcium phosphate (β-TCP) and OCP/Gel. In both cases, radiographic parameters improved, and complete bone union was achieved within six to eight months. OCP/Gel was fully resorbed and replaced by mature bone, while β-TCP remained partially unabsorbed. Both patients recovered uneventfully with stable correction. This is the first clinical report of OCP/Gel use in SPO. OCP/Gel promoted early bone remodeling and complete integration, suggesting it as a safe and effective bone substitute for reconstructive hip surgery.

## Introduction

In pelvic osteotomy for developmental dysplasia of the hip (DDH), bone defects inevitably occur at the osteotomy site, and complete bone filling is desirable to preserve bone stock for potential future hip surgeries [1-3]. Maintaining adequate bone stock not only contributes to long-term joint stability but also facilitates future revision procedures if required. Autologous bone grafting remains the gold standard because it provides osteogenic cells and growth factors essential for bone healing. However, its use is limited by donor-site morbidity and restricted availability [4]. Artificial bone substitutes, such as β-tricalcium phosphate (β-TCP) and hydroxyapatite (HA), are highly biocompatible [5] but lack sufficient osteoinductivity and biodegradability, underscoring the need for bone substitutes with stronger osteogenic potential [6]. Octacalcium phosphate (OCP), which converts into HA in vivo, has been shown to promote osteoblastic differentiation and exhibits excellent osteogenic capacity [7-12]. To further improve its handling and mechanical stability, an octacalcium phosphate/gelatin composite (OCP/Gel; Bricta, Nipro, Osaka, Japan) was developed while maintaining high biocompatibility [13,14]. Preclinical studies have demonstrated that OCP/Gel possesses outstanding biodegradability and promotes early bone regeneration through complete replacement with newly formed bone. We report two cases of DDH treated with spherical periacetabular osteotomy (SPO) using OCP/Gel as a bone substitute.

## Case presentation

Case 1

**Figure 1:**
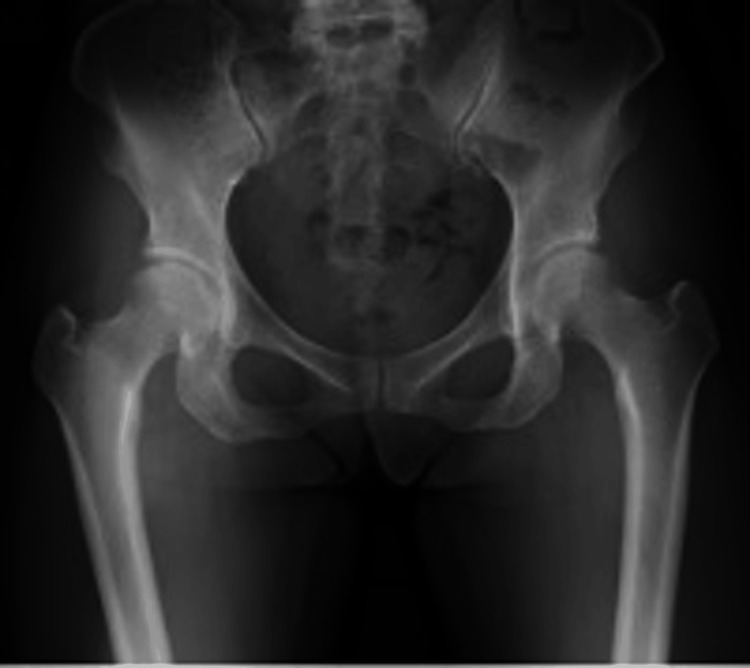
Preoperative radiographs of case 1. CE angle: right 15.4°, left 19.1°; ARO: right 17.8°, left 15.2°; Sharp's angle: right 42.5°, left 40.4°; AHI: right 72.5%, left 72.5%. CE, center–edge; ARO, acetabular roof obliquity; AHI, acetabular head index

A 45-year-old woman developed right hip pain during activity at age 44. Her medical history included anterior cruciate ligament reconstruction, lumbar disc herniation, hysterectomy, and cerebral aneurysm. Independent ambulation was possible, with mild resting and motion pain on the right side. The Japanese Orthopaedic Association (JOA) hip score was 80/100. Preoperative radiographs showed right-sided acetabular dysplasia with center-edge (CE) angle 15.4°, acetabular roof obliquity (ARO) 17.8°, Sharp’s angle 42.5°, and acetabular head index (AHI) 72.5% (Figure 1).

**Figure 2:**
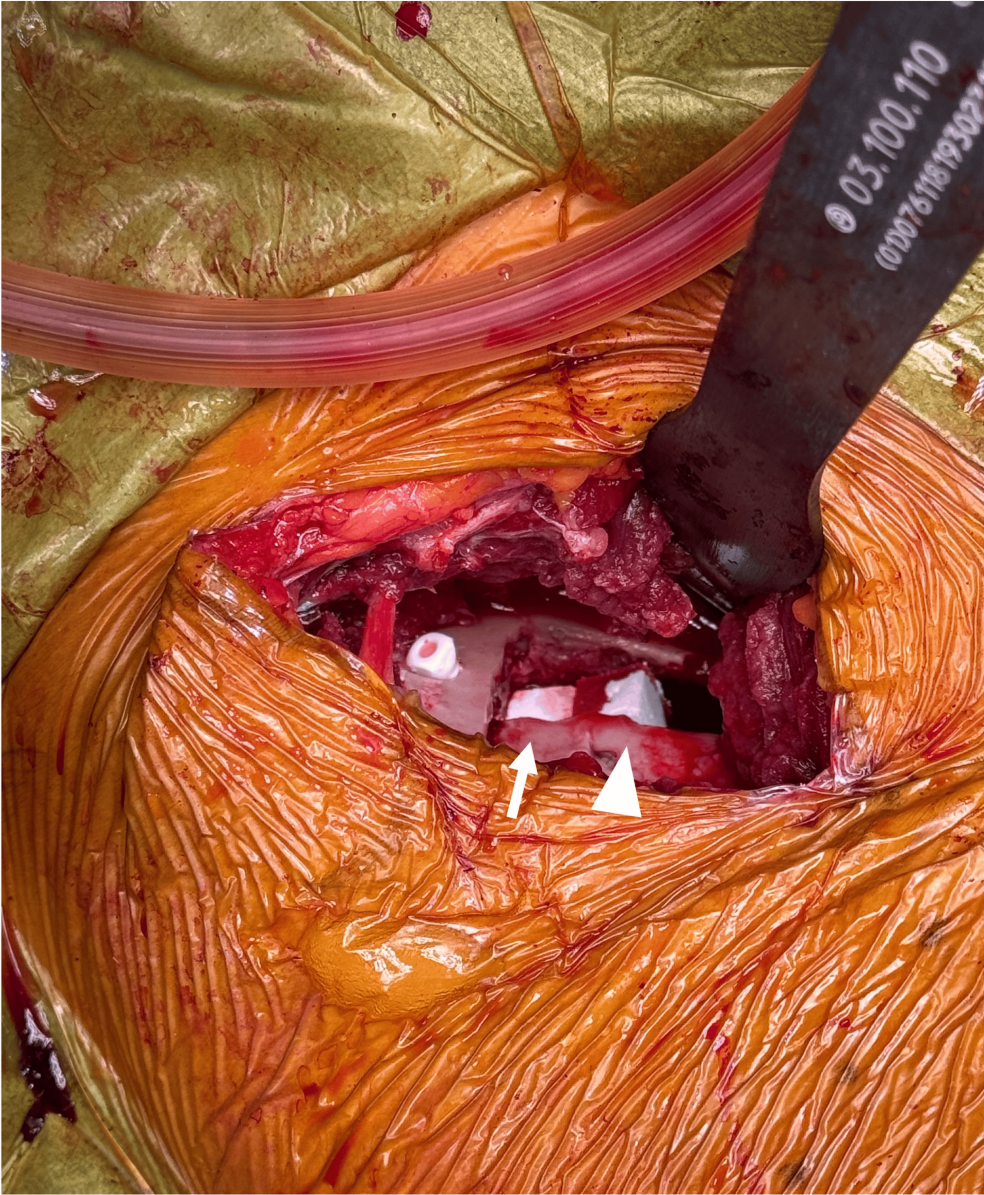
Intraoperative photograph of spherical periacetabular osteotomy in case 1. One β-TCP block (2× 1× 1cm) and three OCP/Gel composites (1× 1× 1cm) were inserted into the osteotomy gap. The white arrow indicates the β-TCP block, and the arrowhead indicates the OCP/Gel composites. OCP/Gel, octacalcium phosphate/gelatin composite; β-TCP, β-tricalcium phosphate

She underwent right SPO via a para-sartorius approach [15], and the reoriented acetabular fragment was stabilized using HA screws. One β-TCP block (2 × 1 × 1 cm) and three OCP/Gel cubes (1 × 1 × 1 cm) were inserted into the osteotomy gap (Figure 2).

**Figure 3:**
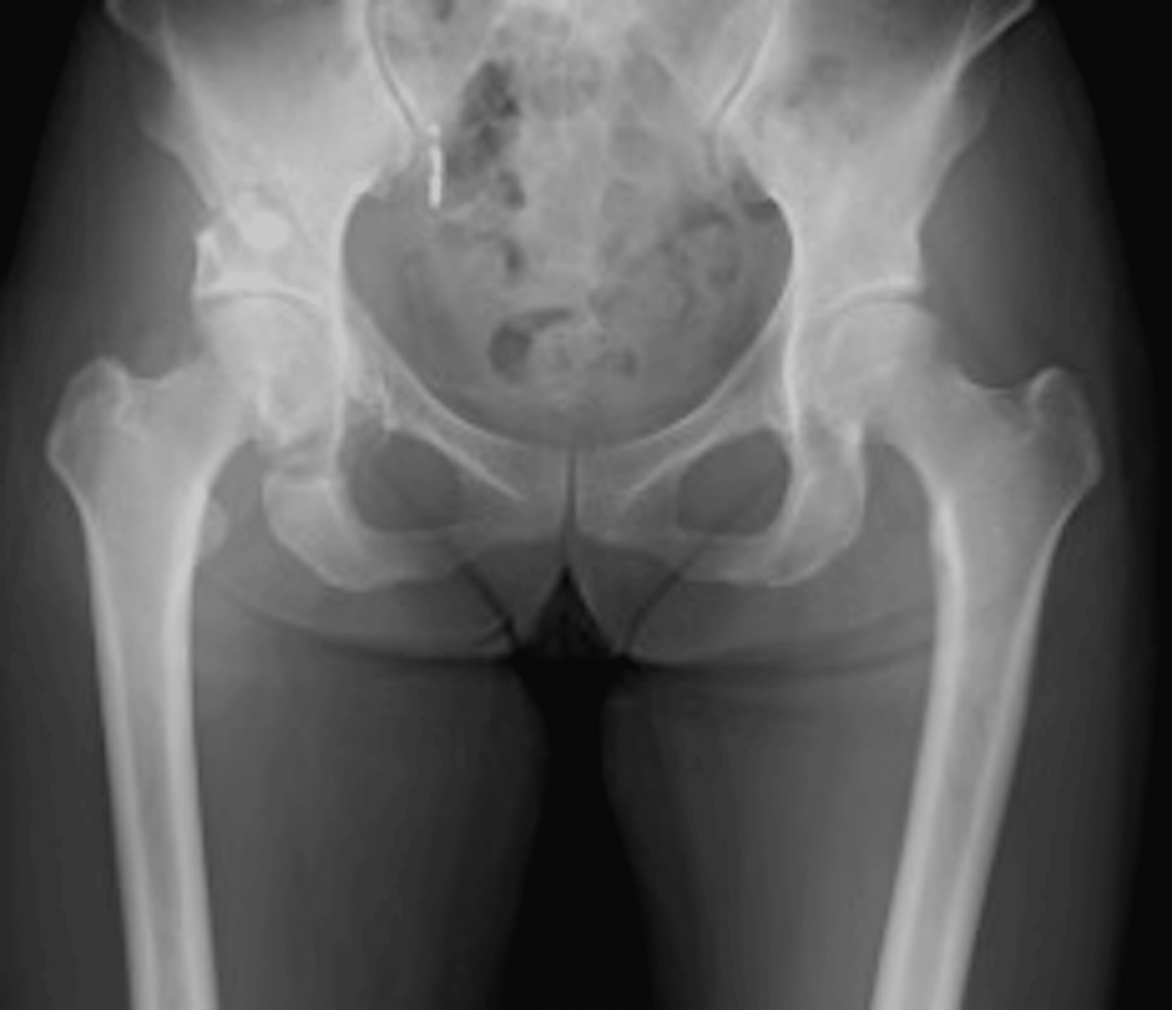
Postoperative radiographs of case 1. CE angle: 29.9°, ARO: 6.6°, Sharp's angle: 52.8°, AHI: 92.3%. CE, center–edge; ARO, acetabular roof obliquity; AHI, acetabular head index

**Figure 4:**
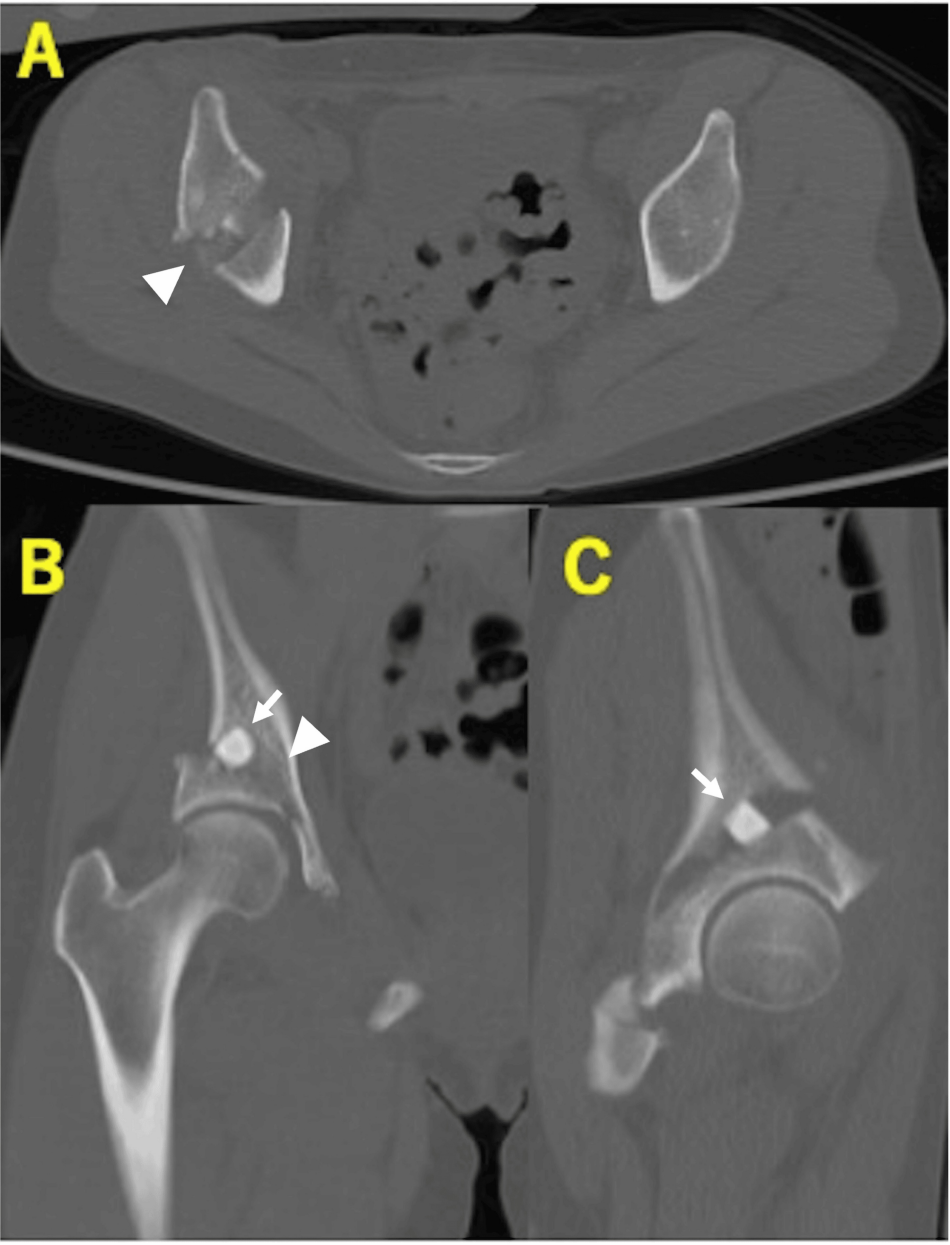
Postoperative computed tomography of case 1. (A) Axial view. (B) Coronal view. (C) Sagittal view. One β-TCP block (2× 1× 1 cm) and three OCP/Gel composites (1× 1× 1 cm) were inserted into the osteotomy gap. The bright white material corresponds to the β-TCP block, while the OCP/Gel is seen as a faint, less radiodense white area. The white arrows indicate the β-TCP block, and the arrowheads indicate the OCP/Gel composites. OCP/Gel, octacalcium phosphate/gelatin composite; β-TCP, β-tricalcium phosphate

Postoperative radiographs demonstrated improved acetabular coverage: CE 29.9°, ARO 6.6°, Sharp’s 52.8°, and AHI 92.3% (Figure 3). CT at three weeks showed that OCP/Gel was not clearly visible within the osteotomy gap (Figure 4).

**Figure 5:**
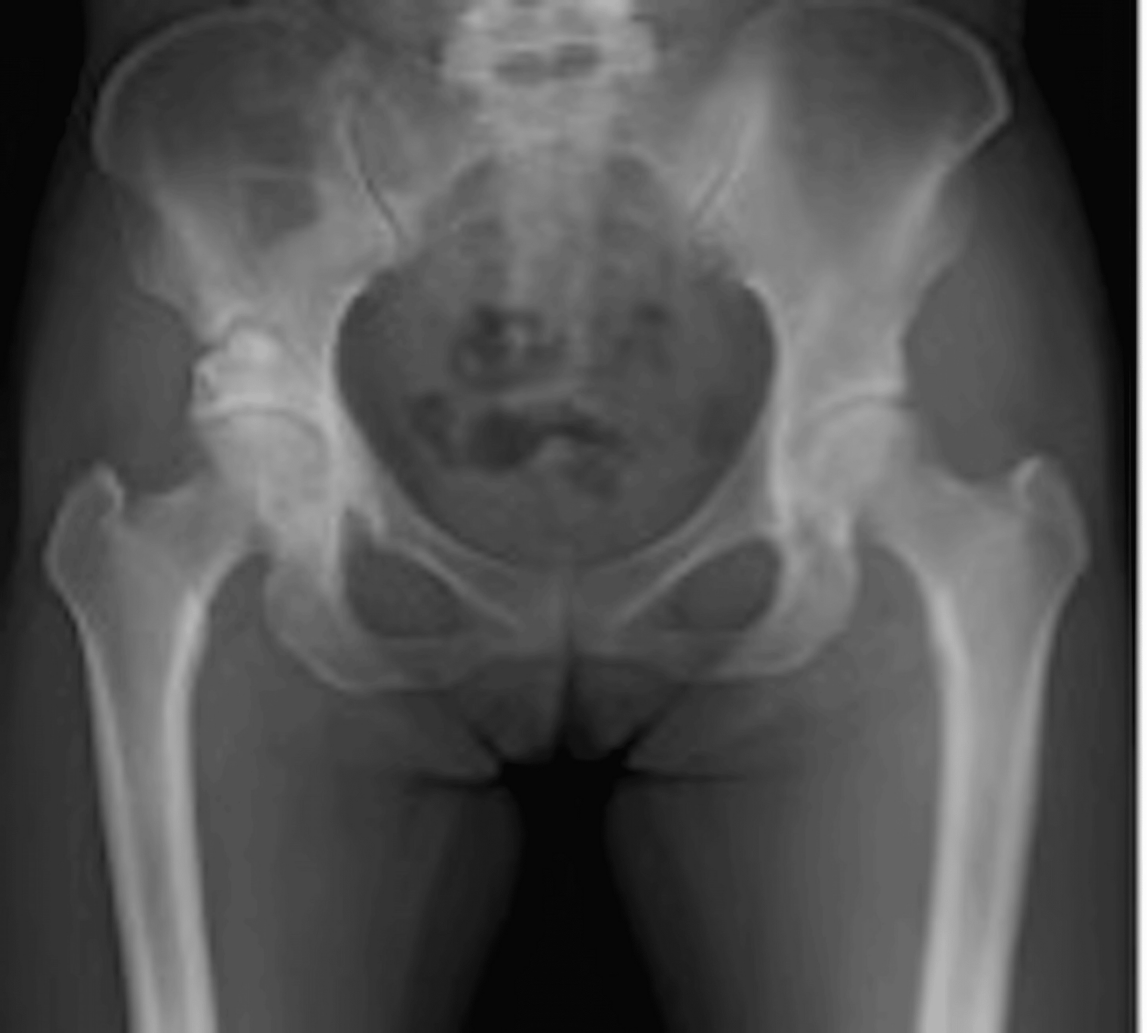
Radiographs at six months postoperatively of case 1. Complete bone union was observed at the osteotomy site. CE angle: 30.4°, ARO: 5.5°, Sharp's angle: 50.3°, AHI: 95.4%. CE, center–edge; ARO, acetabular roof obliquity; AHI, acetabular head index

**Figure 6:**
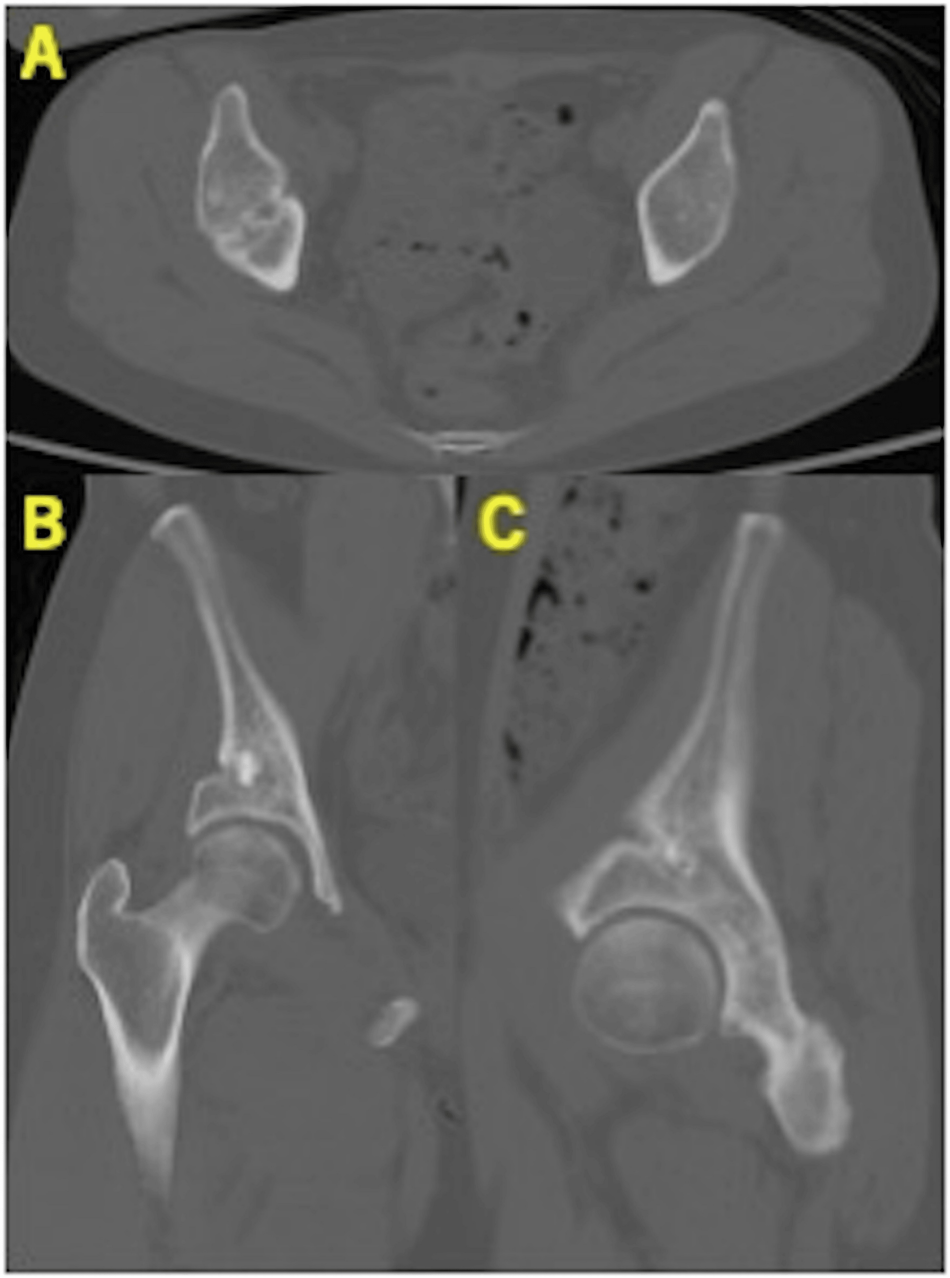
Computed tomography at one year postoperatively of case 1. (A) Axial view. (B) Coronal view. (C) Sagittal view. The β-TCP block remained partially unabsorbed, whereas the OCP/Gel had been completely resorbed and replaced by mature trabecular bone. OCP/Gel, octacalcium phosphate/gelatin composite; β-TCP, β-tricalcium phosphate

Rehabilitation consisted of non-weight-bearing for three weeks, followed by gradual partial weight-bearing from week 3 and full weight-bearing from week 7. At six months, radiographs confirmed solid bone union with minimal correction loss (Figure 5), and at one year, CT demonstrated complete remodeling of the osteotomy site (Figure 6). The β-TCP block was partially absorbed, whereas OCP/Gel was fully resorbed and replaced by mature trabecular bone (Figure 6).

Case 2

**Figure 7:**
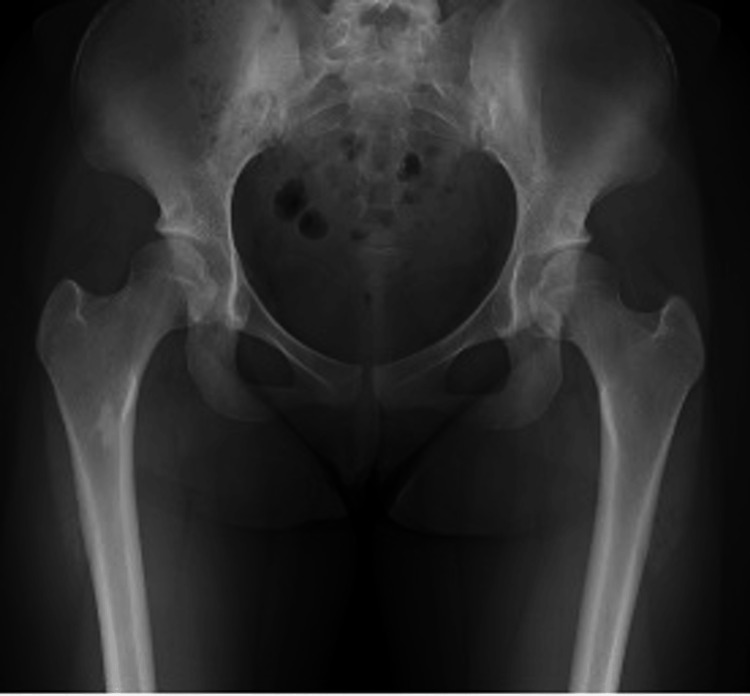
Preoperative radiographs of case 2. CE angle: right 11.6°, left 9.3°; ARO: right 13.7°, left 17.2°; Sharp's angle: right 48.5°, left 48.8°; AHI: right 72.5%, left 71.2%. CE, center–edge; ARO, acetabular roof obliquity; AHI, acetabular head index

A 17-year-old girl who had been diagnosed with DDH in infancy presented with bilateral hip pain during exercise. Her medical history was notable for mild intellectual disability and adjustment disorder. She was able to ambulate independently without assistive devices but experienced bilateral hip pain on movement. Preoperative radiographs showed acetabular dysplasia, which was more severe on the left side: CE 11.6°/9.3°, ARO 13.7°/17.2°, Sharp’s 48.5°/48.8°, and AHI 72.5%/71.2% (Figure 7).

**Figure 8:**
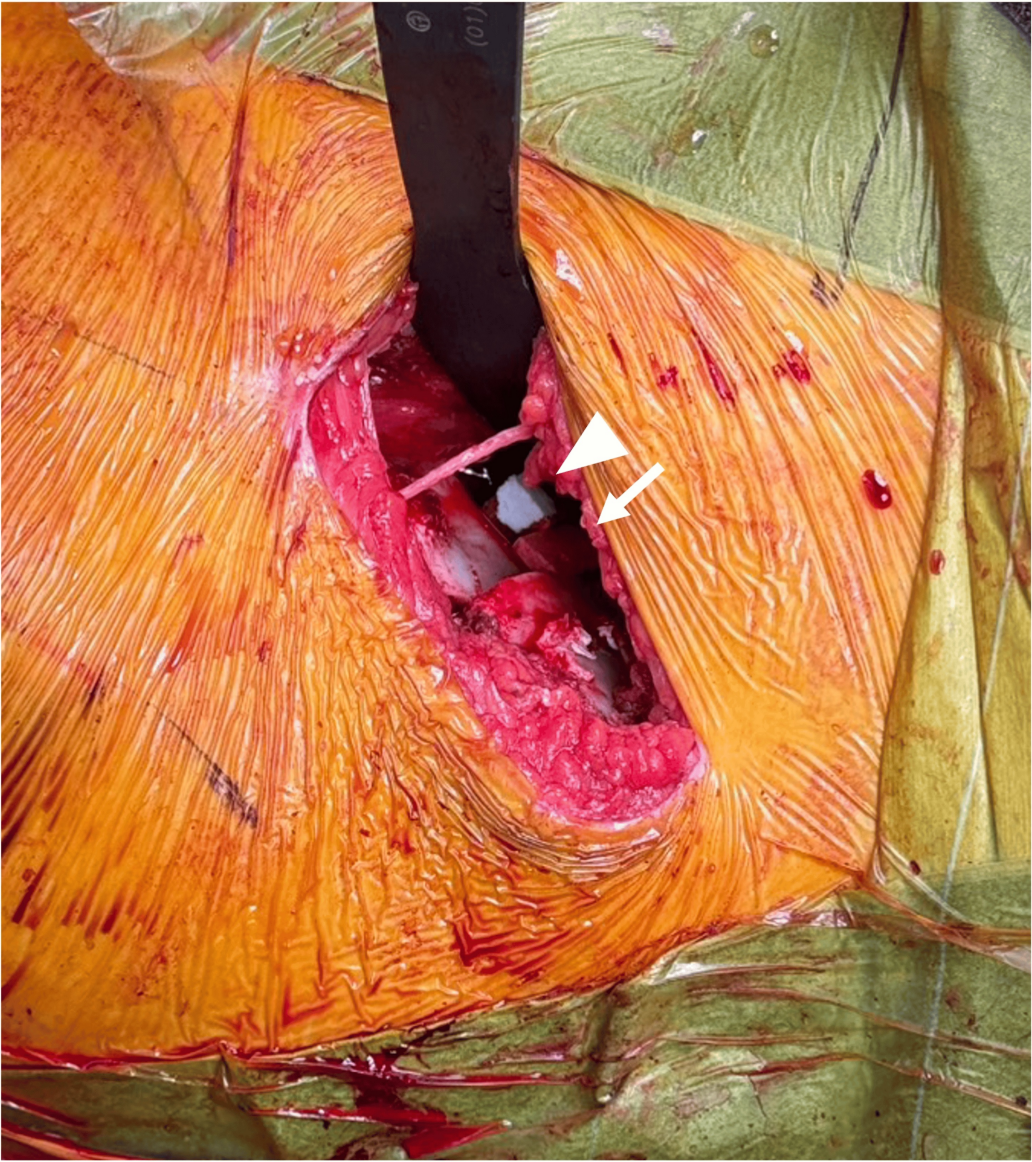
Intraoperative photograph of spherical periacetabular osteotomy in case 2. One β-TCP block (2× 1× 1cm) and four OCP/Gel composites (1× 1× 1cm) were inserted into the osteotomy gap. The white arrow indicates the β-TCP block, and the arrowhead indicates the OCP/Gel composites. OCP/Gel, octacalcium phosphate/gelatin composite; β-TCP, β-tricalcium phosphate

One β-TCP block (2 × 1 × 1 cm) and four OCP/Gel cubes (1 × 1 × 1 cm) were inserted into the osteotomy gap (Figure 8).

**Figure 9:**
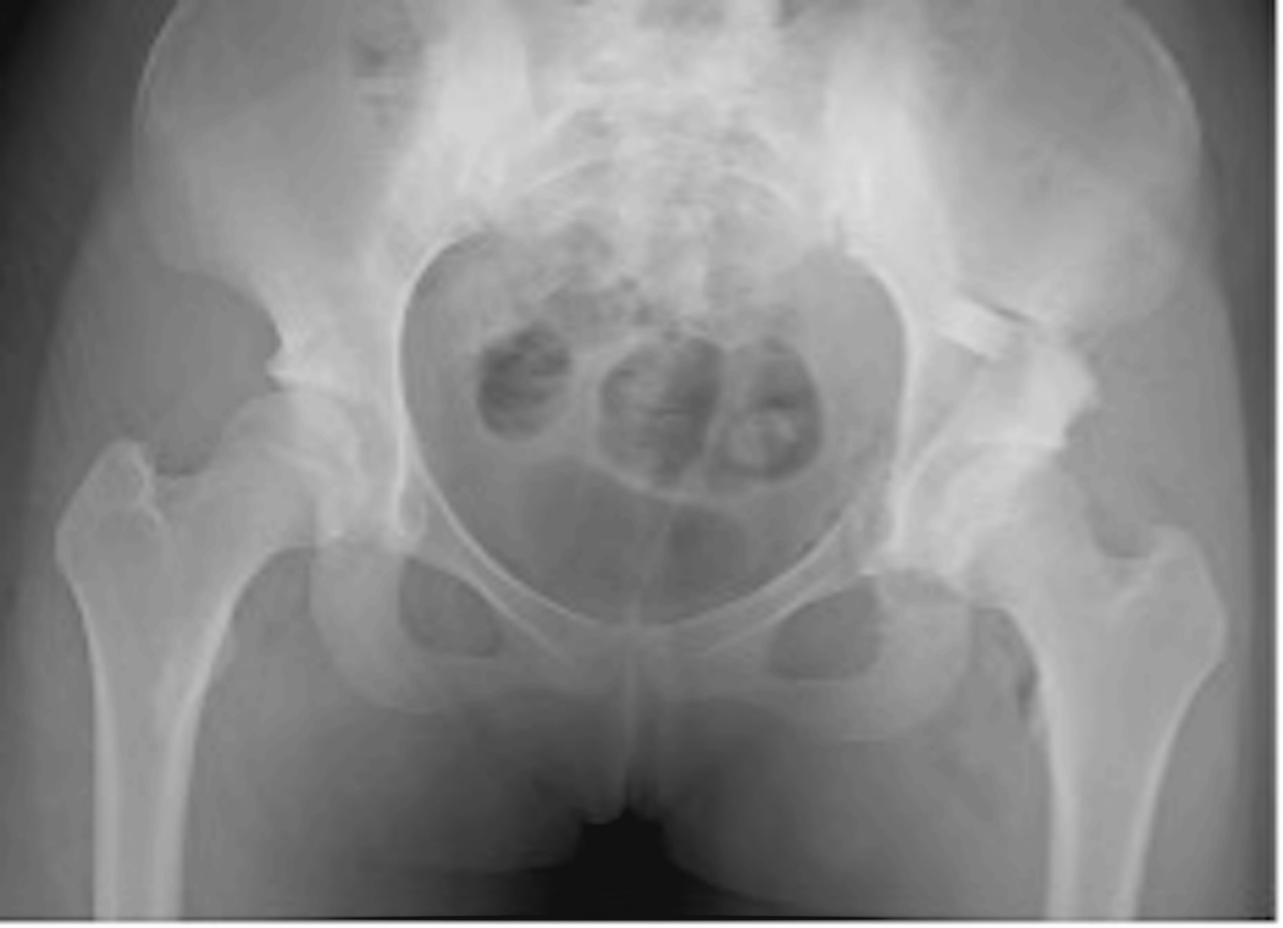
Postoperative radiographs of case 2. CE angle: 25.4°, ARO: 0.3°, Sharp's angle: 51.6°, AHI: 80.0%. CE, center–edge; ARO, acetabular roof obliquity; AHI, acetabular head index

**Figure 10:**
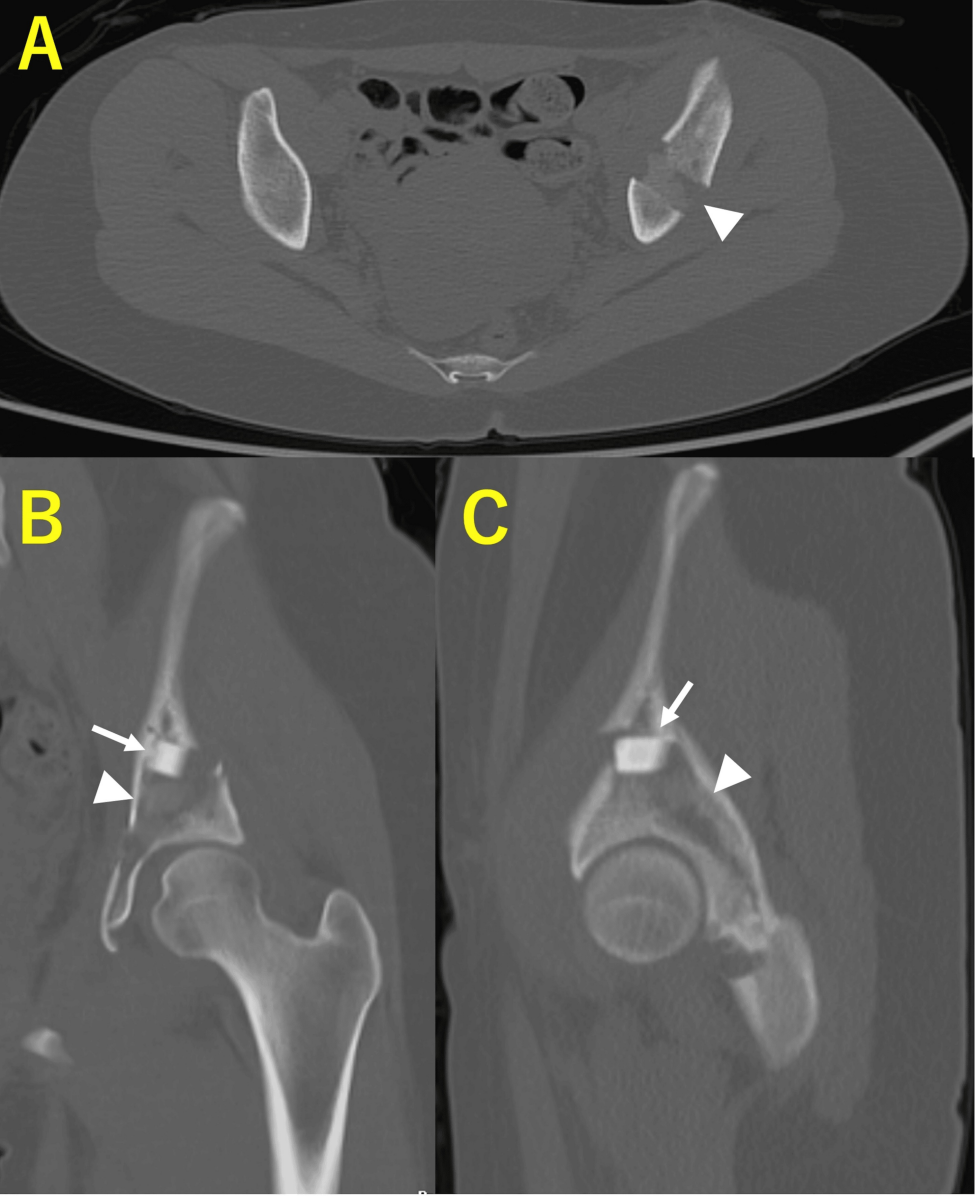
Postoperative computed tomography of case 2. (A) Axial view. (B) Coronal view. (C) Sagittal view. One β-TCP block (2 × 1 × 1 cm) and four OCP/Gel composites (1 × 1 × 1 cm) were inserted into the osteotomy gap. The bright white material corresponds to the β-TCP block, while the OCP/Gel is seen as a faint, less radiodense white area. The white arrows indicate the β-TCP block, and the arrowheads indicate the OCP/Gel composites. OCP/Gel, octacalcium phosphate/gelatin composite; β-TCP, β-tricalcium phosphate

Postoperative radiographs showed improved parameters: CE 25.4°, ARO 0.3°, Sharp’s 51.6°, and AHI 80.0% (Figure 9). CT at three weeks postoperatively revealed that OCP/Gel was not clearly visible (Figure 10).

**Figure 11:**
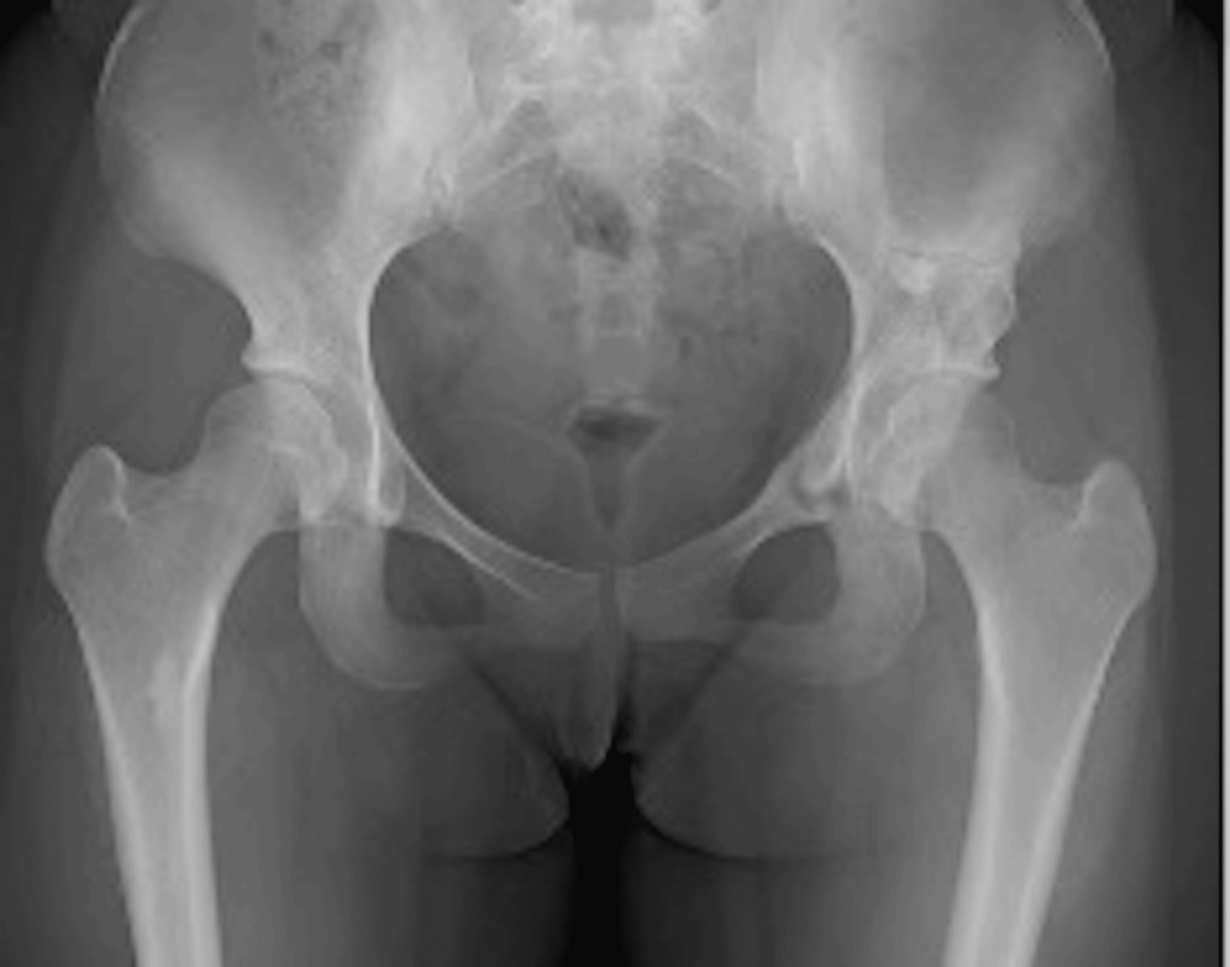
Radiographs at six months postoperatively of case 2. Complete bone union was observed at the osteotomy site. CE angle: 25.0°, ARO: -0.9°, Sharp's angle: 51.1°, AHI: 87%. CE, center–edge; ARO, acetabular roof obliquity; AHI, acetabular head index

**Figure 12:**
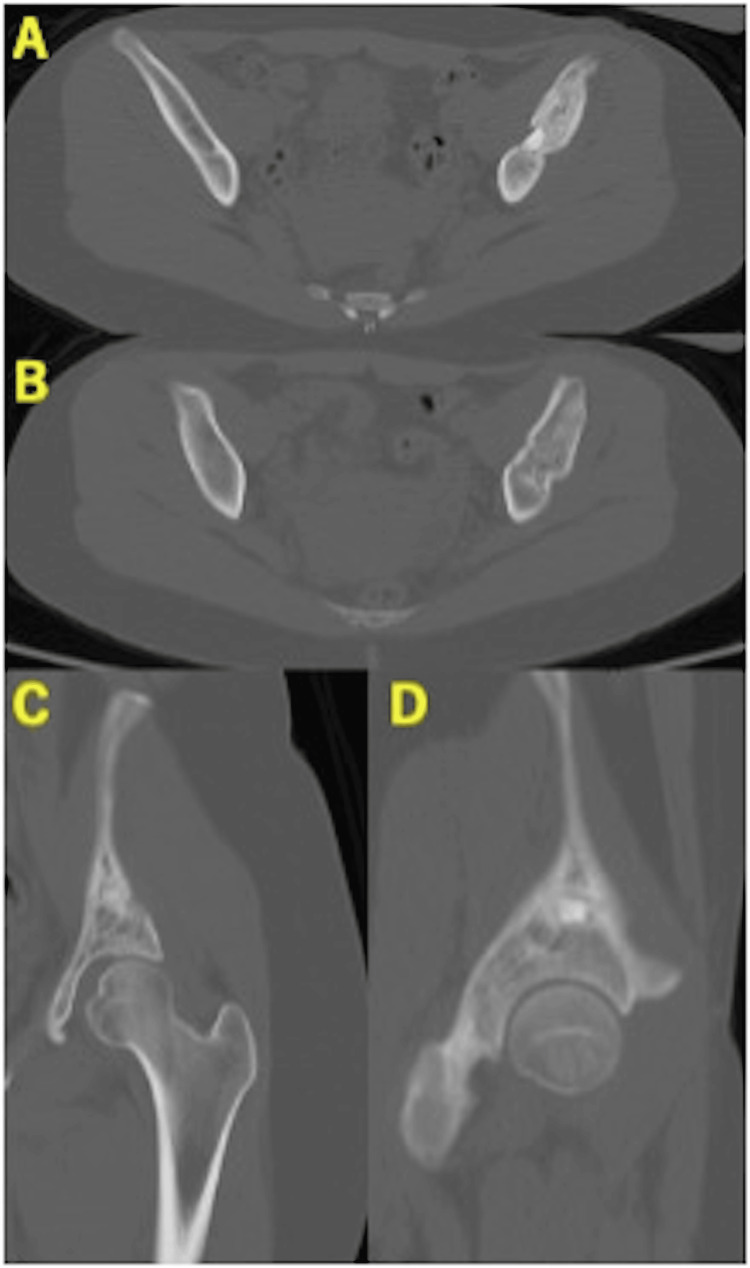
Computed tomography at 7.5 months postoperatively of case 2. (A) Axial view showing the β-TCP insertion site. (B) Axial view showing the OCP/Gel insertion site. (C) Coronal view. (D) Sagittal view. The β-TCP was only partially absorbed, but the OCP/Gel had been completely resorbed and replaced by mature bone trabeculae. OCP/Gel, octacalcium phosphate/gelatin composite; β-TCP, β-tricalcium phosphate

Rehabilitation followed the same staged protocol: non-weight-bearing for the first three weeks, one-third partial weight-bearing from week 3, and full weight-bearing from week 6. At six months, radiographs confirmed bone union, and at seven months, CT demonstrated complete healing at the osteotomy site (Figures 11, 12). The β-TCP block remained partially absorbed, whereas OCP/Gel was completely resorbed and replaced by mature trabecular bone (Figure 12).

## Discussion

Both patients presented with symptomatic acetabular dysplasia and underwent SPO in which a combination of β-TCP and OCP/Gel was used to fill the osteotomy gap. OCP/Gel possesses both osteoconductive and osteoinductive properties, promoting bone regeneration through gradual in vivo conversion to HA [6-12]. In both cases, complete bone union was achieved within six to eight months, during which OCP/Gel was fully resorbed and replaced by mature trabecular bone. In contrast, β-TCP remained partially unabsorbed, a finding consistent with previous reports indicating that OCP accelerates bone remodeling compared with conventional ceramic materials.

β-TCP and HA are known for their biocompatibility and mechanical stability. However, they resorb slowly and show limited osteoinductivity, potentially hindering full remodeling. In contrast, OCP, a physiological precursor of HA, gradually dissolves into Ca-deficient HA, releasing calcium and phosphate ions that enhance osteoblast differentiation and matrix deposition [16]. The complete resorption of OCP/Gel observed here suggests a synchronized remodeling that closely resembles natural bone healing.

The incorporation of gelatin into OCP enhances both handling characteristics and mechanical cohesion, while exposing Arg-Gly-Asp (RGD) motifs that promote integrin-mediated cell adhesion and angiogenesis [13]. Previous studies have demonstrated that OCP/Gel supports vascular invasion and early osteogenesis, findings consistent with the early trabecular and cortical bone formation observed within six months in these cases.

In SPO, reliable filling of the wedge-shaped osteotomy gap is critical for maintaining correction and stability [1-3,15]. The early bone replacement observed with OCP/Gel likely contributed to mechanical stability, thereby reducing the risk of correction loss. The combined use of β-TCP for structural support and OCP/Gel for biological remodeling may provide synergistic advantages in pelvic reconstruction.

A further limitation is that the intrinsic mechanical strength of OCP/Gel is low, and its capacity to function as a load-bearing material in SPO has not been established [14,16]. Therefore, OCP/Gel should be considered primarily a biological adjunct that promotes remodeling rather than a structural graft capable of maintaining correction on its own.

This is the first clinical report describing the use of OCP/Gel in SPO, supporting preclinical findings that highlight its strong osteogenic potential [14,16]. Although the present report is limited by its small sample size and the absence of histological confirmation, the findings suggest that OCP/Gel is a safe, resorbable, and biologically active bone substitute for reconstructive hip surgery. Further studies with larger cohorts and histological evaluation are warranted to optimize its clinical application.

## Conclusions

In this report, two patients with DDH underwent SPO using OCP/Gel as a bone substitute. OCP/Gel was completely resorbed and replaced by mature trabecular bone within six to eight months, achieving solid union and maintenance of correction. In this case, OCP/Gel was associated with radiographic incorporation and maintenance of correction at short-term follow-up. Given its favorable biocompatibility, biodegradability, and osteogenic potential, OCP/Gel may be a safe and effective option to conventional artificial bone substitutes in reconstructive hip surgery, particularly in cases where nonunion is anticipated.
